# Structures and properties of Mg_0.95_Mn_0.01_TM_0.04_O (TM = Co, Ni, and Cu) nanoparticles synthesized by sol–gel auto combustion technique

**DOI:** 10.1039/c8ra00816g

**Published:** 2018-04-17

**Authors:** M. A. Dar, Dinesh Varshney

**Affiliations:** Materials Science Laboratory, School of Physics, Vigyan Bhavan, Devi Ahilya University Khandwa Road Campus Indore 452001 India vdinesh33@rediffmail.com +91-731-2467028 +91-731-2467028

## Abstract

The room temperature structural, optical and dielectric properties of Mg_0.95_Mn_0.05_O and Mg_0.95_Mn_0.01_TM_0.04_O (TM = Co, Ni, and Cu) nanoparticles are reported. All transition metal nanocrystalline samples were successfully prepared by sol–gel auto combustion method. X-ray powder diffraction patterns at room temperature confirmed the formation of single-phase cubic structure with an *Fm*3̄*m* space group for all prepared samples. Slight variation in the lattice parameter of TM doped Mg_0.95_Mn_0.05_O has been observed. Using Rietveld refinement of XRD data, the space group and lattice parameters are determined. Scanning electron microscopy (SEM) measurements were performed to understand the morphology and grain size of the Mg_0.95_Mn_0.01_TM_0.04_O (TM = Co, Ni, and Cu) nanocrystals. The estimated band gaps as calculated by using UV-Vis spectroscopy are found to be 3.59, 3.61, 5.63 and 3.55 eV for Mg_0.95_Mn_0.05_O and Mg_0.95_Mn_0.01_TM_0.04_O (TM = Co, Ni, and Cu) nanocrystals, respectively. Both dielectric constant and dielectric loss is found to decrease due to TM (transition metal) doping. The *ac* conductivity is found to increase with increase in frequency. Electric modulus spectra reflect the contributions from grain effects: the large resolved semicircle arc caused by the grain effect. The results obtained in this study were discussed comparatively with those cited in the literature.

## Abstract 2

The room temperature structural, optical and dielectric properties of Mg_0.95_Mn_0.05_O and Mg_0.95_Mn_0.01_TM_0.04_O (TM = Co, Ni, and Cu) nanoparticles are reported.

## Graphical abstract

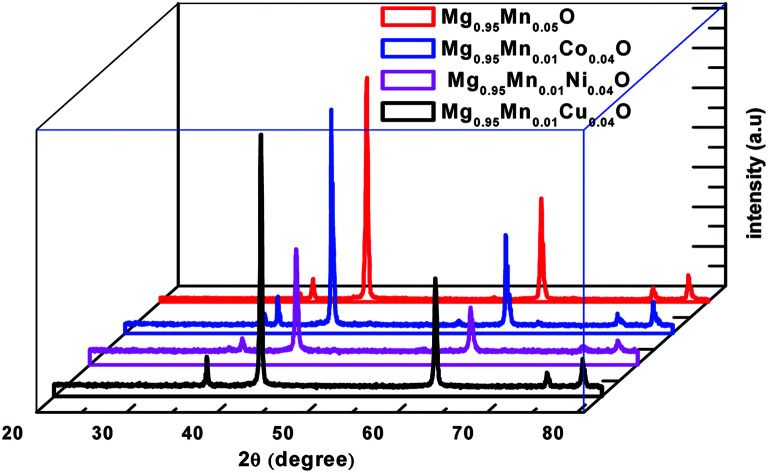

## 1. Introduction

Nanostructures have potential applications in modern science and technology due to their intriguing structural and optical properties.^1,2^ Recently, nanostructures based on oxides have received considerable attention from researchers of the fields of material science, physics and chemistry^3,4^ due to the presence of oxygen, a highly electronegative element, which exhibits the tendency of pulling the bonding electrons towards itself and away from the other elements thereby inducing substantial electric field at the interatomic scale.^5^

Nanomaterials based on metal oxides with high surface to volume ratio have allured considerable interest from the research and scientific community due to their conceivable applications in the field of optoelectronics, nanoelectronics and sensing devices. In particular, magnesium oxide (MgO) is a fascinating basic oxide that has potential applications in catalysis, adsorption, synthesis of refractory ceramics,^6,7^ nano electronics, optoelectronics and sensing devices^8^ and superconductor products.^9^

Adsorption, catalyst supports, and optical sensors are the areas where metal oxides are especially used. Besides these, they are also used in biocompatibility, bioimaging^10^ and many other fields by virtue of their exceptional nanosized structures, superior chemical, morphological and optical band characteristics.^11^ The quantum size effect generated by an increase in the band gap due to a decrease in the quantum allowed state leads to the change in the electrical and optical characteristics of nanosized particles, which in other senses improves the surface and interface effects.^12^

MgO as ceramic has been focused due to its applicability in several areas. MgO is an accepted photocatalyst with exceptional chemical, mechanical, optical and electrical properties. Besides, the inexpensiveness and non-toxicity were regarded as the main reason for the acceptability of MgO materials in modern age of materials. Keeping in view its photocatalytic properties, excellent dielectric properties, the multidimensional applications of MgO such as refractory, paint, translucent ceramics, plasma display panel, absorbent for many pollutants and superconductor products were explored.^13–15^

Magnesium oxide (MgO) exhibits a large band gap of 7.7 eV, excitonic binding energy of ∼80 MeV and posses high transmission in the ultraviolet (Uv) region.^16^ Therefore, MgO can be used to enhance the band gap of ZnO by forming its solid solution with MgO. Since the phase purity, homogeneity, particle size, morphology, as well as crystallinity are the tools to determine the optical properties of materials, the care, control and selection of the method to synthesize the material is of the utmost importance.

A large number of techniques were commonly used for the preparation of MgO powders, such as sol–gel method,^17^ flame spray pyrolysis,^18^ chemical vapour deposition,^19^ co-precipitation method^20^*etc*. Manganese (Mn) enters the MgO crystal preferentially in the divalent charge state occupying cubic Mg sites. Depending on the thermal history of the crystal, higher valence states are possible by virtue of which charge compensation can be achieved by Mg vacancies.^21^ Among the different methods, the sol–gel method is the most effective method to prepare the nanopowders of metal oxides as it is fast, economic and low temperature is required by this method to synthesize the nanosized samples.

The ability to obtain single-phase metal oxide magnetic nanoparticles with controllable particle size and size distribution improves its adequacy in a wide range of technological applications. The sol–gel auto combustion was utilized to synthesize the metal oxide nanoparticles by various researchers in this field. NiFe_2_O_4_ nanoparticles were prepared by a simple and cost-effective polyvinylpyrrolidone (PVP) assisted sol–gel auto-combustion method.^22^ Recently this method also shows option to synthesize advanced spinel ferrite one-dimensional (1D) and two-dimensional (2D) nano-structures.^23,24^ La_0.67_Sr_0.33_MnO_3_ nanoparticles were also successfully synthesized *via* the sol–gel auto-combustion technique.^25^

Herein, the Mg_0.95_Mn_0.05_O and transition metal doped Mg_0.95_Mn_0.01_TM_0.04_O (TM = Co, Ni, and Cu) nanoparticles were prepared by a sol–gel auto combustion method. The structural, optical and electric properties of as prepared powders have been studied by X-ray diffraction (XRD), scanning electron microscopy (SEM), ultra-violet visible spectrum (UV-Vis), Fourier transformation infra red (FT-IR) spectroscopy and dielectric measurements. The main goal of this study is to investigate the effect of TM doping on the structural, vibrational, optical and dielectric properties of Mg_0.96_Mn_0.04_O nanoparticles. For optoelectronic device applications, the controlled band gap is of the utmost importance. In this regard, we made an effort to tune the band gap with different TM doping using UV-Vis spectroscopy.

## 2. Experimental details

### 2.1 Synthesis

The pristine Mg_0.95_Mn_0.05_O and transition metal doped samples of Mg_0.95_Mn_0.01_TM_0.04_O (TM = Co, Ni and Cu) nanoparticles were prepared by sol–gel auto combustion method. All the chemicals were obtained from Merck, India (Analytical grade) and used as such without further purification. The typical synthesis procedure for pure and Co-doped Mg_0.95_Mn_0.05_O is as follows: The aqueous solution of Co doped Mg_0.95_Mn_0.05_O salt were freshly prepared by taking metal nitrates such as magnesium nitrate [Mg (NO_3_)_2_·6H_2_O], manganese nitrate [Mn (NO_3_)_2_·6H_2_O] and cobalt nitrate [Co (NO_3_)_2_·6H_2_O] in appropriate molar ratio. Mg (NO_3_)_2_·6H_2_O were dissolved in 100 ml distilled water and calculated amount of Mn (NO_3_)_2_·6H_2_O and Co (NO_3_)_2_·6H_2_O were added to it. The nitrate salts are favoured as precursors, because they serve as water-soluble low temperature NO_3_^−^ oxidant source for synthesis.

When all the reactants are completely dissolved, citric acid was added to make a metal complex maintaining pH value at 11. The best about the present study is the preparation with the maintenance of pH and citric acid assistance to control reaction and particle size. Citric acid acted as a chelating agent and helps the reaction to proceed. The addition of citric acid dissolved the insoluble residue leading to the formation of a cation–citric acid complex. Further the nitrate salts are favored as precursors, because they serve as water-soluble low temperature NO_3_^−^ oxidant source for synthesis. Many other fuels including dl-alanine, hydrazine, acrylic acid, carbo-hydrazide, ethylene glycol and polyacrylic acid have also shown great promises. The whole solution was stirred through magnetic beet using a magnetic stirrer for 5 hours at 80 °C temperature until the gel was obtained. The gel obtained was dried at 400 °C for 4 hours to remove water and solvent content. The synthesized MgO powder was white in colour. The powder was calcined in air at 600 °C for 10 h and then pressed into pellets of 10 mm diameter with 2 mm thickness. Finally, the pellets were sintered at 600 °C for 6 h. Similar procedure was adopted for the synthesis of all other transition metal doped samples.

The formation of Mg_0.95_Mn_0.05_O takes place according to procedure as follows:
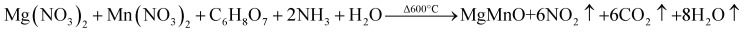


### 2.2 Characterization

The crystal structure, type of phases and particle size of Mg_0.95_Mn_0.05_O and transition metal doped samples of Mg_0.95_Mn_0.01_TM_0.04_O (TM = Co, Ni, and Cu) nanoparticles were investigated by means of room temperature X-ray powder diffraction technique using Bruker D8-Advance X-ray diffractometer with CuKα_1_ (1.5406 Å) radiation. The XRD data were collected in the 2θ range from 10° ≤ 2*θ* ≤ 80° with a step size of 0.02° and a scanning rate of 2°/min. The X-ray generator was set at 40 kV and 40 mA power setting. Scanning electron microscope images were recorded with a Philips XL30 ESEM (environmental scanning electron microscope).

Diffuse reflectance spectra were recorded in the wavelength range 200–850 nm using UV-Vis spectrometer (Perkin Elmer, Lambda 950 – USA to estimate energy band gap). Dielectric measurements were carried out as a function of frequency in the range of 1–10 MHz on Novocontrol alpha-A high performance frequency analyzer at room temperature. High purity silver conducting paste was used to coat on the pellets for better electrical contact for the dielectric measurements.

## 3. Results and discussion

### 3.1 Structural analysis

**Fig. 1:**
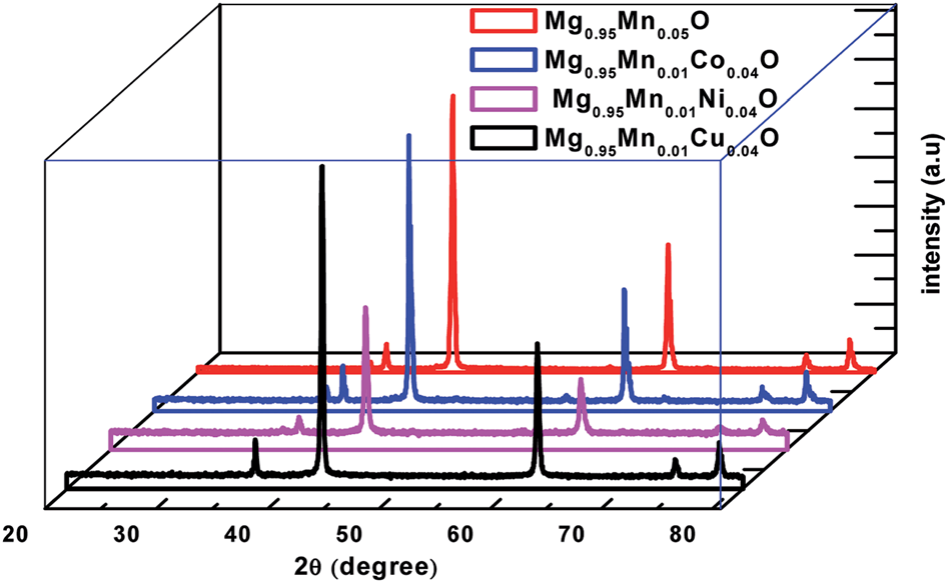
Powder X-ray diffraction pattern of Mg_0.94_Mn_0.06_O and Mg_0.95_Mn_0.01_TM_0.04_O (TM = Co, Ni, and Cu) metal oxide nano particles.

XRD analysis provides information about the structural characteristics of the material as the width and the intensity of the diffraction peaks depend on lattice strain, crystallite size and other imperfections such as stacking faults *etc*. The as prepared Mg_0.95_Mn_0.05_O based transition metal doped powders at the temperature of 600 °C have been structurally characterized by room temperature X-ray powder diffraction (XRD). The XRD patterns of Mg_0.95_Mn_0.01_TM_0.04_O (TM = Co, Ni, and Cu) = (Co, Ni and Cu) samples are shown in Fig. 1 in which all the samples present similar peak positions. The diffraction peaks of samples are indexed as (111), (200), (220), (311) and (222). All the samples exhibit the reflections corresponding to cubic MgO phase having space group *Fm*3̄*m*.

The powders obtained showed a crystallized structures, which is matched with JCPDS PDF (no. 45-946) and consistent with earlier reports.^26,27^ All the samples are in single phase and no diffraction peaks from other species could be detected within measurement range. It means that the TM ion successfully occupies the lattice site rather than interstitial ones. One can observe a slight shift of the position in the diffraction peaks indicating a light variation in lattice parameters. The lattice parameters are calculated using the formula for cubic structure.

1 1/*d*^2^ = *a*/(*h*^2^ + *k*^2^ + *l*^2^)

Here, *d* is the interplanar distance, *h*, *k*, *l* are the miller indices and ‘*a’* is the lattice parameter. One can see the decrease of the lattice parameter from 0.4215 nm in the pristine Mg_0.96_Mn_0.04_O to about 0.42104 nm for Mg_0.95_Mn_0.01_TM_0.04_O. The decrease in the lattice parameters of Mg_0.95_Mn_0.01_Co_0.04_O and Mg_0.95_Mn_0.01_Ni_0.04_O as compared to pristine Mg_0.95_Mn_0.05_O is attributed to the lower ionic size of Co^2+^ (0.72 Å), Ni^2+^ (0.69 Å) and Cu^2+^ (0.72 Å), respectively than ionic radii of Mn^2+^ (80 Å) ion. This is in good agreement with previous reports.^28^

The width of the peak is inversely related to the crystallite size, which has been computed from the full width half maximum (FWHM) of the intense peak using the Debye–Scherer's formula:

2 *d* = 0.9*λ*/*β* cos *θ*

In eqn (2), symbols as ‘*λ*’ is the wavelength of CuKα_1_ radiation and ‘*β’* is the full width half maximum (FWHM) of the highest intense peak of diffracting angle 2*θ*. Table 1 shows the values of particle size (*d*) and lattice variables obtained from the diffraction patterns of the powdered samples of Mg_0.95_Mn_0.05_O and Mg_0.95_Mn_0.01_TM_0.04_O (TM = Co, Ni, and Cu). It was found that the crystallite size of samples lies in the range of 32.3–47.6 nm.

**Table 1:** Structural parameters obtained for Mg_0.95_Mn_0.05_O and Mg_0.95_Mn_0.01_TM_0.04_O (TM = Co, Ni, and Cu) nanocrystals from XRD measurement

Compounds	Space group	Lattice parameters	Particle size (nm)	Optical band gap (eV)
		*a* (Å)		
Mg_0.95_Mn_0.05_O	*Fd*3*m*	4.2150 (4)	32.34	3.59
Mg_0.95_Mn_0.01_Co_0.04_O	*Fd*3*m*	4.2092 (4)	24.00	3.61
Mg_0.95_Mn_0.01_Ni_0.04_O	*Fd*3*m*	4.2106 (4)	34.86	5.63
Mg_0.95_Mn_0.01_Cu_0.04_O	*Fd*3*m*	4.2104 (4)	47.60	3.55

No doubt, particle size is variable with temperature. On annealing the lattice defects and strains generally decreases, however, it can also cause coalescence of crystallites that result in increasing the average size of the nanoparticles. The nano particles of metal oxides in the range of 40–50 nm at around 600 °C are also earlier reported.^29–31^ In this work particle size is calculated using Debye–Scherer's formula: the width of the peak is inversely related to the crystallite size, which has been computed from the full width half maximum (FWHM) of the intense peak. Instrumental broadening is not considered in entire XRD measurement.

**Fig. 2:**
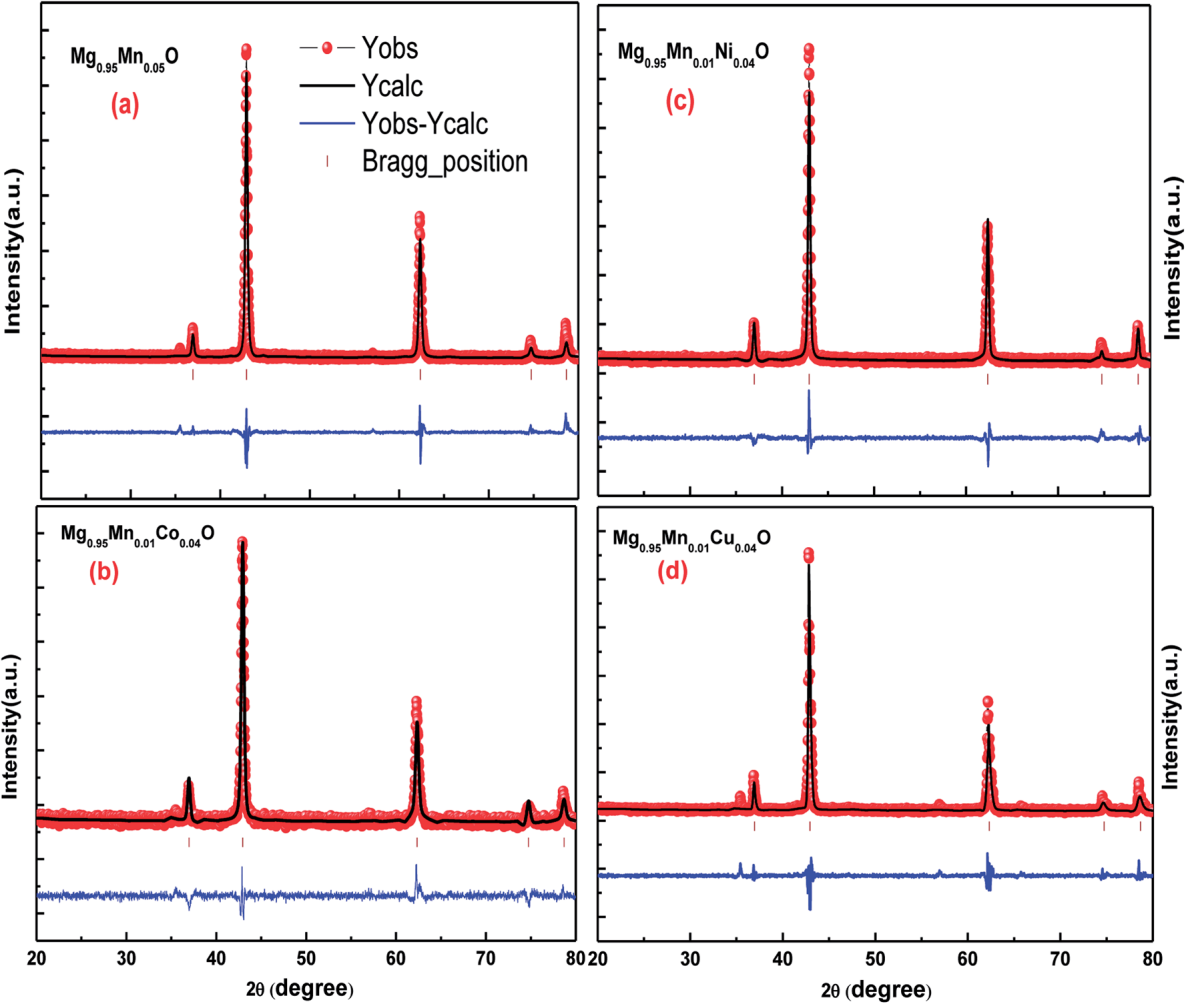
Rietveld refined XRD patterns of Mg_0.94_Mn_0.06_O and Mg_0.95_Mn_0.01_TM_0.04_O (TM = Co, Ni, and Cu) metal oxide nano particles.

Rietveld analyses of the diffraction data collected at the room temperature were carried out using Full Prof refinement program for Mg_0.95_Mn_0.05_O and Mg_0.95_Mn_0.01_TM_0.04_O (TM = Co, Ni, and Cu) nanoparticles. The Pseudo Voigt function is selected to refine the shape of the peak. Background, peak width, peak shape, lattice parameters and atomic positions were refined in the analysis. The Rietveld refined X-ray diffraction (XRD) plots of all the samples under investigation are shown in Fig. 2. Rietveld refined plots further confirms that the pristine Mg_0.95_Mn_0.05_O is in single phase possessing cubic phase structure with *Fm*3̄*m* space group. It is observed that there is no change in the crystal structure due to transition element doping in Mg_0.95_Mn_0.05_O. It indicates that the cubic phase is retained up to 5% doping of Co, Ni and Cu in Mg_0.95_Mn_0.05_O nanoparticles.

The typical values of structural parameters in cubic co-ordinates for all samples refined by a standard Rietveld technique using FullProf refinement program are listed in Table 2 along with the values of the profile factor *R*_p_, weighted profile factor *R*_wp_, expected weighted profile factor *R*_exp_, Bragg factor *R*_B_, structure factor R_F_, goodness of fit *χ*^2^ and goodness of fit (GOF) index. Here, red symbols are the observed profile; the black solid line is the calculated profile, tick marks below indicate the position of the allowed Bragg reflections, the blue line curve at the bottom gives the difference between the observed and calculated data. All these parameters were used as numerical criteria of the quality of the fit of calculated to experimental diffraction data.

**Table 2:** Rietveld refined parameters of Mg_0.95_Mn_0.05_O and Mg_0.95_Mn_0.01_TM_0.04_O (TM = Co, Ni, and Cu) nanocrystals from XRD measurement

Sample Name	Mg_0.95_Mn_0.05_O	Mg_0.95_Mn_0.01_Ni_0.04_O	Mg_0.95_Mn_0.01_Co_0.04_O	Mg_0.95_Mn_0.01_Cu_0.04_O
Space group	*Fm*3̄*m*	*Fm*3̄*m*	*Fm*3̄*m*	*Fm*3̄*m*
*a* (Å)	4.2097 (4)	4.2084 (4)	4.2089 (4)	4.2058 (4)
*V* (Å^3^)	74.6039	74.5359	74.5618	74.6070
*ρ* (g cm^−3^)	4.2097	4.2084	4.2088	4.1254
*R*_p_	25.8	25.8	38.5	37.6
*R*_wp_	24.1	20.2	23.7	30.2
*R*_exp_	13.8	11.00	18.00	13.00
*R*_Bragg_	6.67	3.95	4.31	8.34
*R*_f_	6.85	3.33	2.82	4.54
*GoF*	2	1.8	1.3	2.2
*χ* ^2^	3.036	3.30	1.73	3.7

### 3.2 Microstructural analysis

**Fig. 3:**
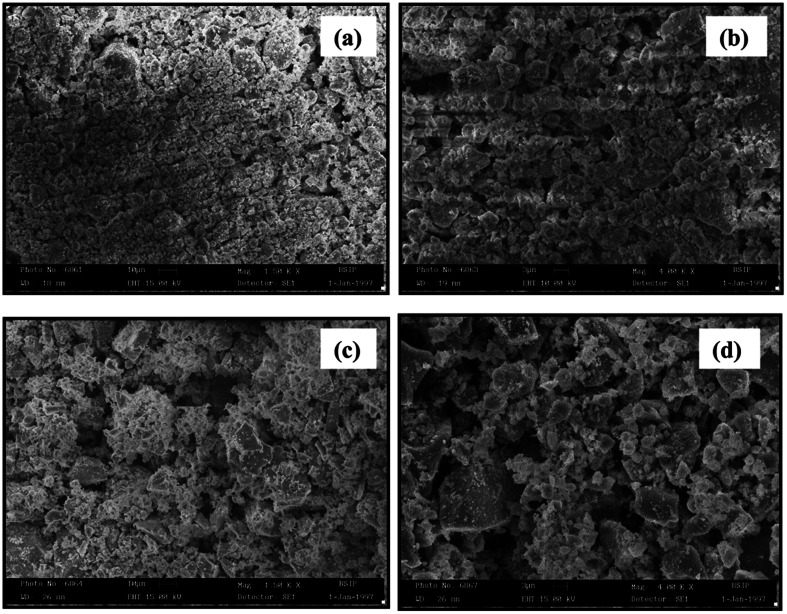
SEM images of Mg_0.94_Mn_0.06_O and Mg_0.95_Mn_0.01_TM_0.04_O (TM = Co, Ni, and Cu).

Fig. 3a–d shows the SEM images of the synthesized Mg_0.95_Mn_0.05_O and Mg_0.95_Mn_0.01_TM_0.04_O (TM = Co, Ni, and Cu) nanocrystals. All the three images illustrate that the synthesized materials are un-agglomerated with spherical morphology. In general, the growing nanocrystals are highly attractive due to large surface energy by virtue of their large surface to volume ratio resulting in the agglomeration of nanocrystals by Ostwald ripening process.

Agglomeration process can be suppressed to control the size of nano crystallites by the introduction of organic molecules during the synthesis process as a capping agent. From SEM images, the average grain size for all four samples under investigation is in the range of 31–48 nm. The crystallite size determined from the SEM measurement is in good agreement with the size as obtained from the XRD.

**Fig. 4:**
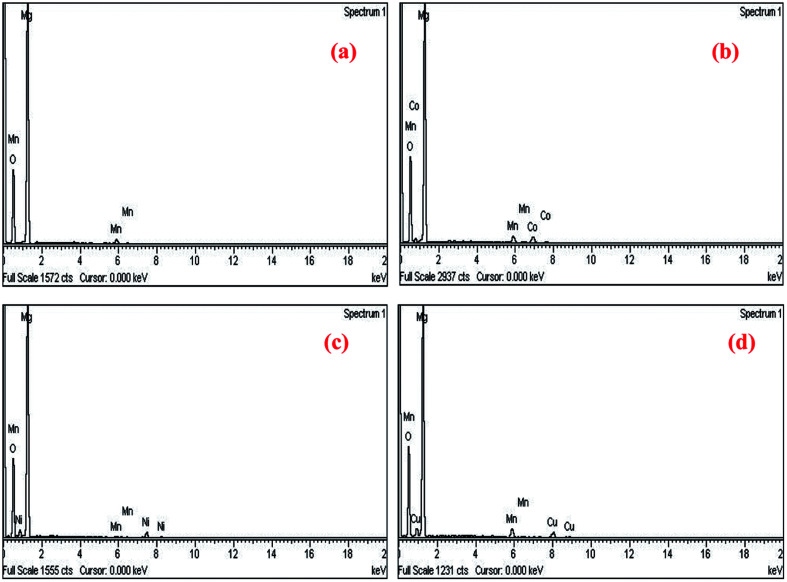
EDAX spectra showing elemental composition of Mg_0.94_Mn_0.06_O and Mg_0.95_Mn_0.01_TM_0.04_O (TM = Co, Ni, and Cu).

In order to confirm the exact composition of the prepared nanocrystals, the EDAX analysis were carried out for Mg_0.95_Mn_0.05_O and Mg_0.95_Mn_0.01_TM_0.04_O (TM = Co, Ni, and Cu) nanocrystals (please see Fig. 4a–d). The EDAX analysis of Mg_0.95_Mn_0.05_O and Mg_0.95_Mn_0.01_TM_0.04_O confirms the presence of Co, Ni and Cu in the Mg_0.95_Mn_0.05_O system and its weight percentage is nearly equal to their nominal stoichiometry within the experimental error.

Thus EDAX spectra show consistency with the experimental concentration used for all the samples. Fig. 4 revealed the presence of Mg, Mn, Co and O as the only elementary components with the absence of any extra element. This indicates that the CoO substitution in the Mg_0.96_Mn_0.04_O do not alter the structure of the host compound which may be attributed to the effective replacement of Mn site by the Co ion.

### 3.3 UV-Vis analysis

UV-visible absorption study is a powerful probe for investigating the effects of impurity doping on optical properties of semiconductor nano structures. The doped nanostructures are expected to have different optical characteristics as compare to pristine nanostructures. The optical diffuse reflectance spectra were recorded using diffuse reflectance spectroscopy to determine the optical band gaps of the as synthesized samples. All spectra were recorded in the range of 200–800 nm.

In order to calculate the direct band gap, Tauc relation is used:

3 (*αhυ*) = *A*(*hυ* − *E*_g_)^*n*^

**Fig. 5:**
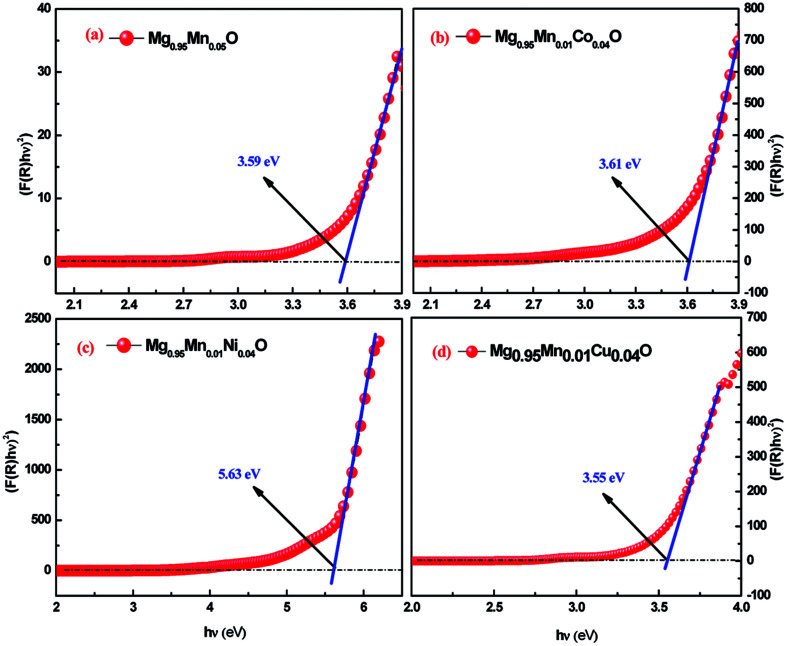
UV-Visible spectra of Mg_0.94_Mn_0.06_O and Mg_0.95_Mn_0.01_TM_0.04_O (TM = Co, Ni, and Cu) nanopowders obtained at room temperature.

In eqn (3), the notations *α* is the absorption coefficient, *A* is a constant, *n* = 1/2 for direct band gap semiconductor. The *E*_g_ values are determined by extrapolating the linear region of the (*hυF*(*R*))^2^ as functions of *hυ*. In other words, the *hυ* value of *x*-axis at (*hυF*(*R*))^2^ = 0 gives the band gap (*E*_g_). Fig. 5 shows the plot for the percentage of reflection (*hυF*(*R*))^2^ as a function of band gap energy *hυ* (eV) for all the studied samples. An extrapolation of the linear region of a plot of (*αhν*)^2^*vs. hν* gives the value of the optical band gap *E*_g_ (Fig. 5).

The estimated band gaps were 3.59, 3.61, 5.63 and 3.55 eV for Mg_0.95_Mn_0.05_O and Mg_0.95_Mn_0.01_TM_0.04_O (TM = Co, Ni, and Cu) nanocrystals, respectively. The calculated value of optical band gap is found to vary from 3.59 eV for Mg_0.95_Mn_0.05_O to 3.55 eV for Mg_0.95_Mn_0.01_Cu_0.04_O. The band gaps of Co and Ni doped samples are greater than pristine Mg_0.95_Mn_0.05_O material. However, Cu doped sample has lower band gap than Mg_0.95_Mn_0.05_O. This suggests that decrease in the band gap of Cu-doped Mg_0.95_Mn_0.05_O nanoparticles is due to the incorporation of Cu into Mg_0.95_Mn_0.05_O matrix which alters the electronic structure leading to the appearance of intermediate energy level.^32^

*E*_g_ values of MgMnO nanoparticles increased with Ni content. The incorporation of Ni is accompanied by a systematic high-energy shift of the band gap extending down to the blue spectral range. The increase in the band gap or blue shift can be explained on the basis of the Burstein–Moss effect about filling the bottom of the conduction band depending on the increase in the carrier concentration. Therefore, the interstitial of Ni^2+^ in MgO lattice may cause an increase in the carrier concentration and the Fermi level moves closer to the conduction band with an increase in the carrier concentration. Consequently, the filling of the conduction band by electrons generally causes an increase in the optical band gap or blue shift. The same increase in *E*_g_ was also earlier reported.^33^ They reported a blue shift of the absorption edges from 3.22 eV (undoped ZnO) to 3.30 eV (5% Co-doped ZnO). Such an increase in the optical band gap is consistent with previous observations.^34–36^

### 3.4 Dielectric measurement

The dielectric constant and dielectric loss of a material are two basic criteria that a material must match for the better applicability and efficiency as they affect many optoelectronic and transport properties. Dielectric studies have been done on Mg_0.95_Mn_0.05_O and Mg_0.95_Mn_0.01_TM_0.04_O (TM = Co, Ni, and Cu) nanocrystals to investigate any variation of dielectric constant and dielectric loss with frequency and different transition metal ion doping.

The dielectric properties of materials are characterized by the complex dielectric constant (*ε*) which is represented by *ε = ε*′ − *jε*′′. The real part (*ε*′) of dielectric constant is the measure of the amount of energy stored in a dielectric due to the applied field and the imaginary part (*ε*′′) of dielectric constant describes the dissipated energy in dielectric. The value of real part of dielectric constant (*ε*′) is calculated by using *ε = Ct*/(*Aε*_0_) where *ε*_0_ is the permittivity of free space, *t* is the thickness of pellet, *A* is the cross sectional area and *C* is the capacitance of pellet.

**Fig. 6:**
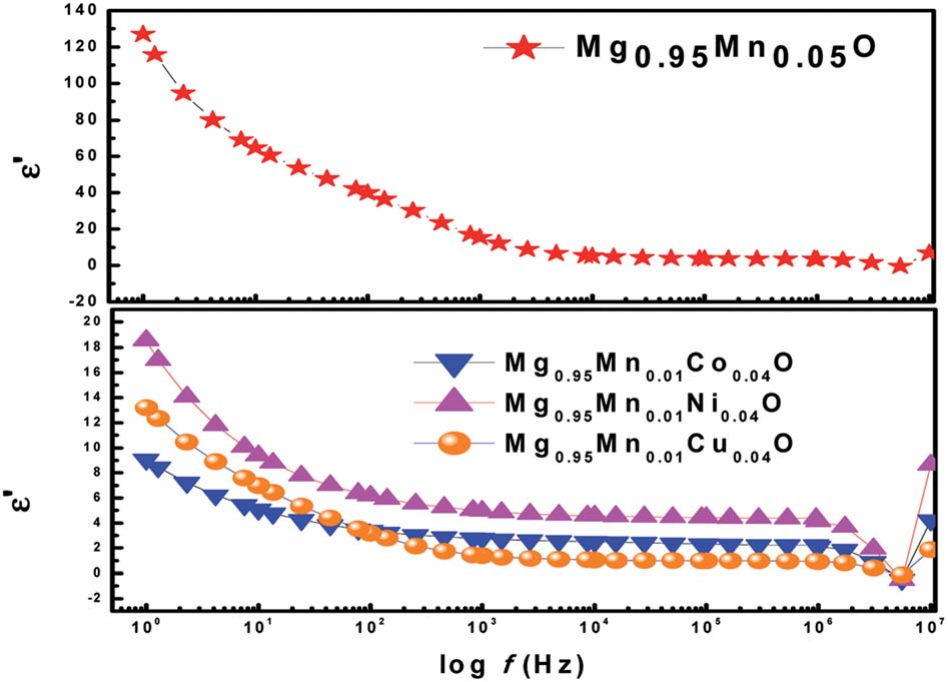
Variation of real part of dielectric constant with frequency of Mg_0.94_Mn_0.06_O and Mg_0.95_Mn_0.01_TM_0.04_O (TM = Co, Ni, and Cu) at room temperature.

Fig. 6 shows the variation of dielectric constant (*ε*′) with frequency for Mg_0.95_Mn_0.05_O and Mg_0.95_Mn_0.01_TM_0.04_O (TM = Co, Ni, and Cu) at room temperature. At lower frequency the dispersion of dielectric constant was observed. The large value of dielectric constant at lower frequency observed is attributed to the grain boundary defects or the presence of oxygen vacancies.^37^ In addition to that, the large value of the dielectric constant is also due to the fact that the nanoparticles of Mg_0.95_Mn_0.05_O under the application of electric field act as nano dipoles. With the decrease in the size of nano particle, the particles per unit volume increases and thereby increases dipole moment per unit volume and hence the high dielectric constant.^38^ It is noticed that the dielectric constant is decreased in TM doped Mg_0.95_Mn_0.05_O as compared with pure Mg_0.95_Mn_0.05_O. The dielectric constant decreased in the whole applied frequency region with TM dopant up to 4%. Because, the polarization is decreased due to the formation of grains surrounded by insulating grain boundaries. The doping may create many defects into MgO, thus reduces the dielectric constant.

However, in the higher frequency regime *i.e*. frequency above 0.5 MHz, ε′ is independent of frequency. As beyond a certain frequency limit, the hopping between different metal ions cannot follow the changing field. Further, dielectric constant of Mg_0.95_Mn_0.05_O decreases due to Co, Ni and Cu doping at Mn-site. The lowest dielectric constant is observed for Mg_0.95_Mn_0.01_Co_0.04_O which is ∼9 at lower frequency and highest dielectric constant is obtained for pristine Mg_0.95_Mn_0.05_O of about 128.

**Fig. 7:**
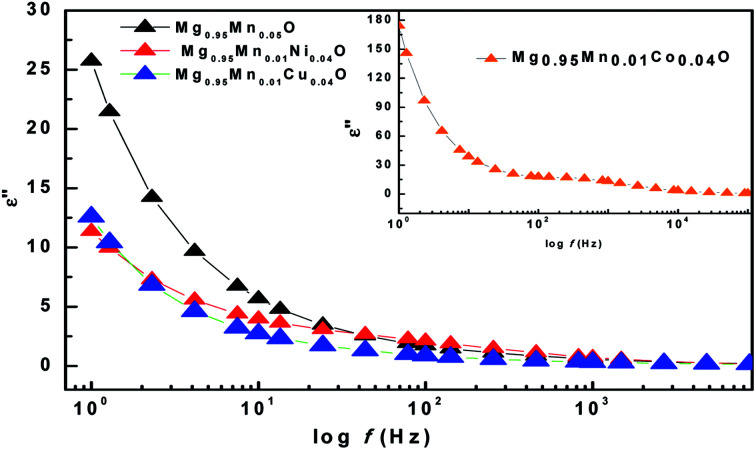
Variation of imaginary part of dielectric constant with frequency of Mg_0.94_Mn_0.06_O and Mg_0.95_Mn_0.01_TM_0.04_O (TM = Co, Ni, and Cu) at room temperature.

The imaginary part (*ε*′′) of dielectric constant of Mg_0.95_Mn_0.05_O and Mg_0.95_Mn_0.01_TM_0.04_O (TM = Co, Ni, and Cu) nanocrystals shows a normal dielectric behaviour as observed earlier.^39^ We note that the imaginary part of dielectric constant (*ε*′′) shows a decreasing trend with increase in frequency (see Fig. 7), almost similar to real part of dielectric constant and loss tangent. This variation in imaginary part of dielectric constant (*ε*′′) with respect to frequency may be due to several factors; such as conduction mechanism (hopping of electron between Mn^3+^ and Mn^2+^), materials composition of sample, annealing temperature, grown technique and particle size.^40^

**Fig. 8:**
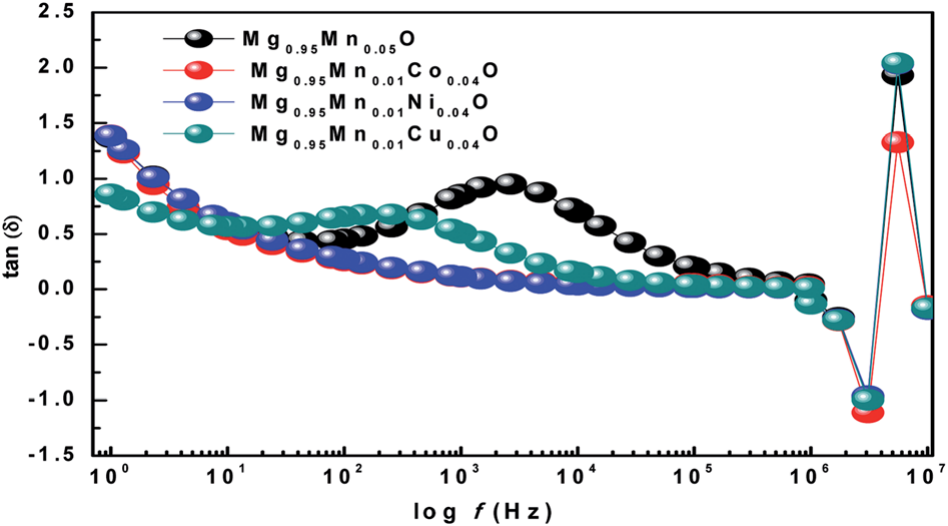
Variation of Dielectric Loss with frequency of Mg_0.94_Mn_0.06_O and Mg_0.95_Mn_0.01_TM_0.04_O (TM = Co, Ni, and Cu) at room temperature.

The ratio of energy dissipated and energy stored in the material determines the dielectric loss factor (tan *δ*) and variation of dielectric loss with frequency at room temperature is shown in Fig. 8. It shows that the dielectric loss (tan *δ*) decreases with the increase of frequency in all the samples. Wide peaks are observed in Mg_0.95_Mn_0.05_O and Mg_0.95_Mn_0.01_Cu_0.04_O samples which are believed to exist due to the resonance between the hopping frequency of charge carriers and applied frequency. No peak like behaviour is observed in Mg_0.95_Mn_0.01_Co_0.04_O and Mg_0.95_Mn_0.01_Ni_0.04_O samples as their resonance frequency lies beyond the measurement frequency range.^41^

In this work, we have observed that dielectric loss decreases at higher frequency which is due to suppression of domain wall motion. The dielectric loss is maximum at lower frequencies and is due to nearly equal hopping frequency between different ionic sites and the frequency of the applied field. Dielectric loss of the doped samples Mg_0.95_Mn_0.01_Co_0.04_O, Mg_0.95_Mn_0.01_Ni_0.04_O and Mg_0.95_Mn_0.01_Cu_0.04_O is slightly less than Mg_0.95_Mn_0.05_O, which is due to the decrease in dielectric constant as, described earlier.

**Fig. 9:**
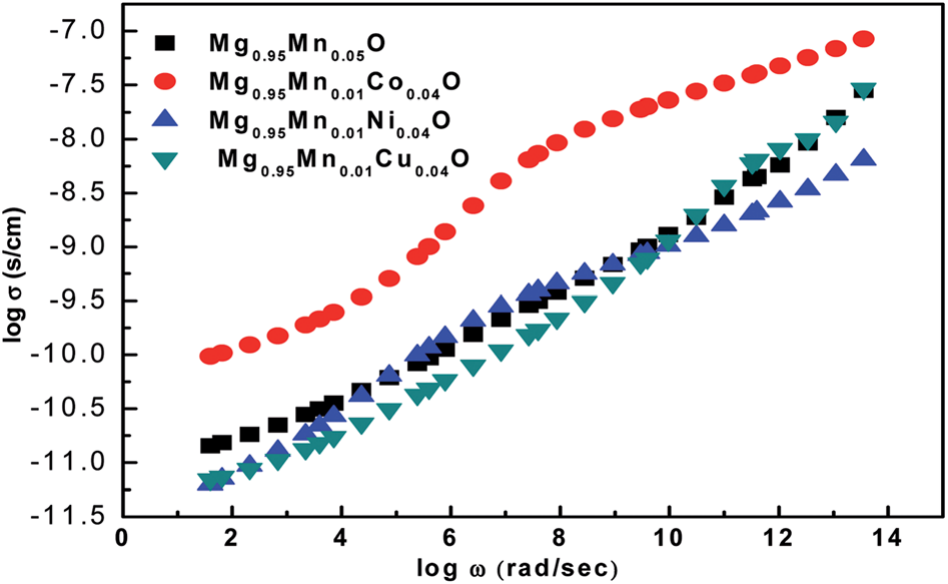
Variation of *ac* conductivity as a function of frequency for Mg_0.94_Mn_0.06_O and Mg_0.95_Mn_0.01_TM_0.04_O (TM = Co, Ni, and Cu) at room temperature.

The *ac* conductivity of pure Mg_0.95_Mn_0.05_O and Mg_0.95_Mn_0.01_TM_0.04_O (TM = Co, Ni, and Cu) nanoparticles is shown in Fig. 9. It is found that the *ac* conductivity progressively increases with the increase in the frequency of the applied *ac* field. This is because rising in frequency would improve the electron hopping frequency. The *ac* conductivity is initially high for pure Mg_0.95_Mn_0.05_O and found to be less in transition metal doped Mg_0.95_Mn_0.01_TM_0.04_O (TM = Co, Ni, and Cu) nanoparticles. The substitution of transition metal doping may initiate the defect ions, oxygen vacancies in the Mg_0.95_Mn_0.05_O nanoparticles and tends to segregate at the grain boundaries due to the diffusion at the time of sintering and cooling processes. These defects block the flow of charge carriers at the grain boundaries and cause decreases in the conductivity initially thereafter the conductivity of doped nanoparticles increases. Mg_0.95_Mn_0.01_Co_0.04_O has highest value of *ac* conductivity both at low and high frequencies as compared to other transition metal doped samples.

**Fig. 10:**
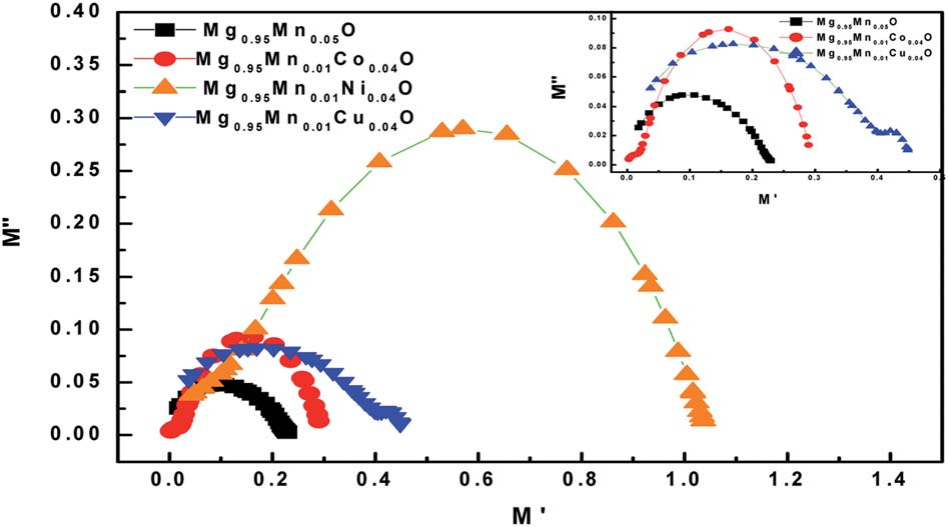
*Cole*–*Cole* plot for Mg_0.94_Mn_0.06_O and Mg_0.95_Mn_0.01_TM_0.04_O (TM = Co, Ni, and Cu) at room temperature.

The dielectric modulus plots of *M*′′ (= *ε*′′(*ω*)/[*ε*′(*ω*)^2^ + *ε*′′(*ω*)^2^]) as functions of *M*′ (=*ε*′(*ω*)/[*ε*′(*ω*)^2^ + *ε*′′(*ω*)^2^]) for various composition is shown in Fig. 10. Inset of Fig. 10 shows low frequency region modulus plot due to the grain boundary contribution. The large semicircle obtained in Mg_0.95_Mn_0.01_Co_0.04_O is believed to be induced by the grain effect, due to the smaller capacitance value dominated in the electric modulus spectra. On the other hand, the small semicircle obtained in Mg_0.95_Mn_0.05_O, Mg_0.95_Mn_0.01_Co_0.04_O and Mg_0.95_Mn_0.01_Cu_0.04_O might be attributed to the grain boundary effect. With the huge difference (orders of magnitude) between the resistive values of grains and grain boundaries, it is difficult to obtain two full semicircles for grains and grain boundary on the same scale in the impedance plot. Complex modulus analysis is suitable when materials have nearly similar resistance but different capacitance.^42^

## 4. Conclusions

In summary, we have synthesized Mg_0.95_Mn_0.05_O and Mg_0.95_Mn_0.01_TM_0.04_O (TM = Co, Ni, and Cu) nanocrystalline samples using sol–gel auto combustion method. The structural, microstructural, optical, and dielectric properties were sensitively dependent on the incorporation of TM^2+^ ions into MgO matrix. X-ray diffraction (XRD) confirmed the single-phase cubic structure with *Fm*3̄*m* space group for all prepared samples.

The calculated values of optical band gap vary from 3.59 eV for Mg_0.95_Mn_0.05_O to 3.55 eV for Mg_0.95_Mn_0.01_Cu_0.04_O. The lowest dielectric constant is observed for Mg_0.95_Mn_0.01_Co_0.04_O which is ∼9 at lower frequency and highest dielectric constant is obtained for pristine Mg_0.95_Mn_0.05_O of ∼128. Dielectric loss (tan *δ*) of Mg_0.95_Mn_0.05_O and Mg_0.95_Mn_0.01_Cu_0.04_O nanostructured materials decreases with increase in frequency having wide peaks in certain range of frequency, which is due to the resonance among the hopping frequency of charge carriers and applied frequency. Electric modulus spectra reflect the contributions from grain effects: the large resolved semicircle arc caused by the grain effect.

## Conflicts of interest

There are no conflicts to declare.

## Supplementary Material
